# Vitamin A and Hydrochlorothiazide Causing Severe Hypercalcemia in a Patient With Primary Hyperparathyroidism

**DOI:** 10.1177/2324709618823805

**Published:** 2019-01-11

**Authors:** Ron T. Varghese, Khaled Khasawneh, Raman K. Desikan, Anandaraj Subramaniam, Todd Weaver, Ganesh K. V. Nair

**Affiliations:** 1 White River Medical Center, Batesville, AR, USA; 2 CHI Little Rock Diagnostic Clinic, Little Rock, AR, USA

**Keywords:** nutrition, vitamin A, primary hyperparathyroidism

## Abstract

*Objective*. To report a case of severe hypercalcemia, exacerbated by vitamin A supplementation and hydrochlorothiazide, in a patient with primary hyperparathyroidism. *Methods*. Clinical and laboratory findings are presented along with response to therapy. *Results*. A 68-year-old white female presented to the emergency department complaining of nausea, vomiting, and altered mental status. Laboratory findings revealed calcium 15.8 mg/dL (8.4-10.2), albumin 4.1 g/dL (3.8-4.8), and parathyroid hormone 62 pg/mL (14-64). Serum calcium improved after intravenous hydration with normal saline. Prior to this hospitalization, over-the-counter medications were significant for calcium (600 mg daily), vitamin A (11 000 IU daily), and vitamin D (800 IU daily).The patient’s prescription medications were significant for hydrochlorothiazide (12.5 mg daily). Twenty-four-hour urine calcium was subsequently found to be 146 mg (35-250). Myeloma, lymphoma, and sarcoidosis were ruled out as the etiology for hypercalcemia. The diagnosis of primary hyperparathyroidism was confirmed. She was treated surgically for primary hyperparathyroidism. The right and left superior parathyroid showed hypercellular parathyroid on pathology. The patient was normocalcemic after surgery. *Conclusion*. Previous reports suggest that very high doses of vitamin A is required to cause hypercalcemia. This case suggests that in a setting of primary hyperparathyroidism and hydrochlorothiazide therapy, vitamin A may contribute to the development of severe hypercalcemia in patients who are on calcium and vitamin D supplements. Given their biologic effects, public awareness needs to be created regarding the injudicious use of vitamins.

## Introduction

Vitamin supplementation is commonly used, and most of these supplements are available over-the-counter. We present a case of severe hypercalcemia, exacerbated by vitamin A supplementation and hydrochlorothiazide (HCTZ), in a patient with primary hyperparathyroidism (HPT).

## Case Report

A 68-year-old white female presented to emergency department with complaints of nausea, vomiting, and altered mental status.

One day prior, she presented to an outpatient clinic with left lower quadrant pain and diagnosed with clinical diverticulosis. She was started on oral ciprofloxacin and metronidazole from the clinic. She started taking these medications that afternoon, both on an empty stomach. She began to have dizziness and vomiting since 4 am on the morning of presentation. She was brought by Emergency Medical Service to the emergency department that afternoon.

The patient was afebrile (97.3°F) but had elevated blood pressure at presentation (176/107 mm Hg). Physical examination revealed that she had dry oral mucosa, her abdomen was soft, with no organomegaly, and she did not have any neurological deficits. Laboratory findings on admission are shown in Table 1. Having nausea and vomiting, physical examination with findings of dry mucus membranes and laboratory tests with elevated blood urea nitrogen and creatinine suggested dehydration. She was also noted to have hypercalcemia (Table 1). Hypercalcemia by itself could likely cause volume depletion, since hypercalcemia leads to diuresis and vasoconstriction and contributes to acute kidney injury. However, in this patient, given her recent history of vomiting, it would be difficult to attribute volume depletion solely to hypercalcemia. There was no history of primary HPT, thyroid disease, or thyroid cancer in her family. She was treated with intravenous hydration with normal saline. Serum calcium reduced from 15.8 to 13.6 mg/dL with hydration alone. Prior to this hospitalization, she was on over-the-counter calcium 600 mg, vitamin D_3_ 800 IU, vitamin A 11 000 IU daily, for history of macular degeneration, and valsartan/HCTZ 320 to 12.5 mg daily for her hypertension; all medications had been taken for 3 years. These medications were stopped during hospitalization.

**Table 1.** Laboratory Findings on Admission.

	Value	Reference Range
Calcium	15.8 mg/dL	8.4-10.2
Albumin	4.1 g/dL	3.8-4.8
PTH	62 pg/mL	14-64
25-OH vitamin D	46 ng/mL	30-100
WBC	6.9 k/uL	4.5-11
Hemoglobin	12.5 g/dL	12.0-16.0
Sodium	140 mmol/L	137-145
Potassium	3.6 mmol/L	3.5-5.1
Chloride	99 mmol/L	98-107
Bicarbonate	29 mmol/L	21-32
BUN	35 mg/dL	7-18
Creatinine	2.6 mg/dL	0.55-1.02
Total bilirubin	0.40 mg/dL	0.2-1.0
AST	18 U/L	15-37
ALT	23 U/L	12-78
Alkaline phosphatase	103 U/L	46-116
Total protein	8.7 g/dL	6.4-8.2
Globulin	4.6 g/dL	2.2-4.4

Abbreviations: PTH, parathyroid hormone; 25-OH, 25-hydroxy; WBC, white blood cells; BUN, blood urea nitrogen; AST, aspartate aminotransferase; ALT, alanine aminotransferase.

She was subsequently referred for endocrinology evaluation. One week later, laboratory findings in endocrinology clinic showed elevated serum calcium and parathyroid hormone (PTH; Table 2). 25-Hydroxy (25-OH) vitamin D; 1,25-OH vitamin D; and PTH-RP were within normal limits. Serum immunofixation electrophoresis showed poorly defined area of monoclonal protein. She underwent oncology evaluation to rule out multiple myeloma.

**Table 2.** Laboratory Findings at Weeks of Follow-up^a^.

	Week 1	Week 2	Week 4
Calcium	15.8 mg/dL (8.4-10.2)	11.1 mg/dL (8.6-10.5)	10.9 mg/dL (8.6-10.5)
Albumin	4.1 g/dL (3.8-4.8)	4.4 g/dL (3.6-4.7)	3.9 g/dL (3.6-4.7)
Creatinine	2.6 mg/dL (0.55-1.02)		0.8 mg/dL (0.8-1.4)
Phosphorus		3.0 mg/dL (2.5-4.8)	3.2 mg/dL (2.5-4.8)
PTH	62 pg/mL (14-64)	98.9 pg/mL (14-64)	48.6 pg/mL (14-64)
PTH-RP		15 pg/mL (14-27)	
25-OH vitamin D	46 ng/mL (30-100)	55.8 ng/mL (30-100)	
1,25-OH vitamin D		45 pmol/L (19-72)	

Abbreviations: PTH, parathyroid hormone; PTH-RP, parathyroid hormone–related protein; 25-OH, 25-hydroxy.

a Reference range for labs in parenthesis.

Laboratory findings on oncology evaluation showed that urine Kappa light chains were increased, as was the urine free Kappa/Lambda ratio, but no monoclonal spike was detected in urine electrophoresis. On quantitative immunoglobulin (Ig) evaluation, IgG (1327 mg/dL) and IgA (258 mg/dL) were normal, and IgM minimally elevated (289 mg/dL). Immunofixation revealed IgG Lambda monoclonal protein. She thus had monoclonal gammopathy of undetermined significance but no myeloma; therefore, myeloma was not contributing to hypercalcemia. Normal 1,25-dihydroxyvitamin D level and angiotensin converting enzyme level ruled out the possibility of granulomatous diseases like sarcoidosis and lymphoma.

She came for review in endocrinology clinic, a few weeks after her initial visit. She had been off HCTZ for a few weeks at the time of this visit. Laboratory findings in this visit (Tables 2 and 3) revealed elevated calcium, with non-suppressed PTH, and normal 24-hour urine calcium. The diagnosis of primary HPT was thus confirmed.

**Table 3.** Laboratory Findings at Week 7.

Laboratory Findings (24-Hour Urine), Volume 2700 mL	Value	Reference Range
Calcium	146 mg	35-250
Creatinine	1.27 g	0.8-2.8
Creatinine clearance	134.5 mL/min	88-128

She was subsequently treated surgically for primary HPT. The right and left superior parathyroid showed hypercellular parathyroid on pathology. The patient was normocalcemic after surgery. The calcium on follow-up visit about a week after surgery was 9.8 mg/dL (8.5-12.1).

## Discussion

Primary HPT is a fairly common condition (7/1000 adults); however, severe hypercalcemia secondary to primary HPT is uncommon. Hypercalcemia of primary HPT can be exacerbated by coexisting conditions, which cause increase in bone turnover. These conditions causing increased bone turnover include Paget’s disease and immobilization.^1,2^ HCTZ decreases urinary excretion of calcium and thus may also have contributed to the hypercalcemia.^3,4^ Although ciprofloxacin and metronidazole were started 1 day prior to the development of hypercalcemia, to the best of our knowledge, there is no known association between hypercalcemia and ciprofloxacin or metronidazole. It is also possible that vitamin A and calcium may also have affected the management of diverticulitis, since they decrease the absorption of quinolones including ciprofloxacin.^5^ Hypervitaminosis A is a rare cause of hypercalcemia.^6,7^ Vitamin A is thought to act directly on bone to stimulate osteoclastic resorption and/or inhibit osteoblastic formation, and thus cause hypercalcemia. The recommended dietary allowance for vitamin A in women aged more than 14 years is 700 µg RAE (retinol activity equivalents) or 5000 IU daily. Reports suggest that very high doses of vitamin A is required to cause hypercalcemia. Acute toxicity has been reported to occur at doses of 25 000 IU/kg, while chronic toxicity can occur at 4000 IU/kg daily for 6 to 15 months.^8^ The case suggests that in a setting of primary HPT and HCTZ therapy, even smaller doses of vitamin A can lead to severe hypercalcemia. Hammoud et al have reported hypercalcemia secondary to hypervitaminosis A (7000 IU daily) in patients with chronic kidney disease.^9^ This patient was also on calcium and vitamin D replacement. This may also have contributed to worsening of the hypercalcemia, as has been reported in earlier studies.^10^ Gallagher et al^11^ have reported that episodes of hypercalcemia and hypercalciuria occur commonly in patients on calcium and vitamin D supplementation, and this was unrelated to the dose of vitamin D dose or serum 25-OH vitamin D. This case teaches that a commonly used antihypertensive diuretic and vitamin supplements may sometimes lead to life-threatening situations given the right setting.

## Conclusion

This case suggests that in the clinical setting of HPT, volume depletion, and HCTZ treatment, vitamin A may contribute to the development of severe hypercalcemia in patients who are on calcium and vitamin D supplements. Further studies are warranted in this regard to elucidate the specific role of vitamin A in contributing to severe hypercalcemia in the backdrop of primary HPT.

We should try to improve awareness in the population that vitamins have biological effects, and the injudicious use of over-the-counter vitamins and supplements may lead to serious health issues, triggered by certain clinical conditions, especially in the elderly with multiple comorbidities. Physicians should also be aware that overuse of vitamins may also cause atypical presentation of diseases.
